# Four Matters of Interpretation: The Constitutional Phenomenon in Comparative Studies

**DOI:** 10.1093/ojls/gqaf002

**Published:** 2025-02-17

**Authors:** Ming-Sung Kuo

**Keywords:** comparative constitutional law/studies, constitutional comparison as interpretation, comparative turn, constitutional phenomenon, myth of scientism, positivist trap

## Abstract

This article takes a close look at the state of comparative constitutional studies as constitutional scholarship is taking a comparative turn. It first surveys the field and identifies four varieties – doctrinal, law-and-society, documentary, and cultural – of constitutional comparison and then critically investigates the state of comparative constitutional studies. Through this two-stage engagement, this article aims to make two main analytical points. First, at the core of each of the four varieties of comparative constitutional studies lies an interpretive exercise oriented by its distinctive purpose. Second, the social sciences’ growing influence on constitutional comparison has entailed a myth of scientism in the field, which may inadvertently impoverish comparative constitutional studies as a whole. It concludes with a cautionary note on the comparative turn in studying constitutional ordering. With its prevalent focus on formal institutions and norms in constitutional orders, the comparative turn may unwittingly limit studies of the multifaceted constitutional phenomenon.

## 1. Introduction: Making Sense of the Constitutional Phenomenon

In 2009, William Twining delivered his Montesquieu Lectures, ‘Globalisation and Legal Scholarship’, at Tilburg University in the Netherlands.^1^ In his lectures, Twining predicted a new comparative law informed by a ‘global perspective’ to be thriving in future legal scholarship,^2^ but was less sanguine about the role of comparative law in constitutional studies.^3^ Yet, five years later, a ‘renaissance’ of comparative constitutional law was declared.^4^ That renaissance has since continued. Today, ‘comparative’ becomes the marker of good constitutional studies, while constitutional scholarship untinged by comparison is facing more and more questioning.

As constitutional scholarship is seen as taking a ‘comparative turn’,^5^ some fundamental questions about comparative constitutional studies are still left unasked. Does ‘comparative’ always move in tandem with the constant pursuit of better knowledge of constitutional orders? Could the comparative approach (inadvertently) take constitutional scholarship backward to the latter’s traditional territory that has been virtually defined by the (positive) law? Is it not inconceivable that comparative studies might impoverish rather than enrich our understanding of constitutional ordering? What should comparative constitutional studies look like so that the multifaceted constitutional phenomenon can be properly grasped?^6^

In this article, I take up these questions about the state of constitutional scholarship in light of the diverse use of the comparative approach. As suggested above, constitutional studies, regardless of the approach and standpoint, are aimed at better understanding of norms, institutions, and other phenomena in relation to constitutional ordering. Seen in this light, the enterprise of making sense of the constitutional phenomenon inevitably involves the activity of interpreting.^7^ At the core of comparative constitutional studies,^8^ I shall argue, is a matter of interpretation.^9^ Yet, the exercise of interpretation is oriented by its purpose.^10^ Where purpose multiplies, we see matter*s* of interpretation.

Through a survey of the diverse use of the comparative approach in constitutional studies, I aim to show that comparative constitutional studies can be classified into four varieties in terms of purpose: doctrinal, law-and-society (LS), documentary and cultural.^11^ By bringing to the fore the distinct purposes of these four varieties of constitutional comparison, I take the measure of the field of comparative constitutional studies. In this way, I wish to make an analytical point: comparative constitutional studies should be conducted in a way that the distinctive purpose of the comparison can be served. With the activity of interpreting at the core of comparative constitutional studies brought to light, epistemic and cognitive diversity in constitutional comparison and the comparativist’s mission to make sense of the complex constitutional phenomenon can be better appreciated. Instead of a symbolic gesture towards fellow comparativists in the constitutional field, ‘methodological pluralism’ and ‘analytical eclecticism’ are necessitated by the object of their study itself—the complex and multifaceted phenomenon of constitutional ordering. Suggestions of intellectual diversity in constitutional comparison should not only be trumpeted in theory, but also reflected in the practice of comparative constitutional studies.^12^

Mindful that the *raison d’être* of comparative constitutional studies is to enrich studies of constitutional orders without narrowing our understanding of the constitutional phenomenon, I further strike a cautionary note on the state of comparative constitutional studies: the lure of scientism as suggested in the drive for universality and comprehensiveness in enlarging the ‘family’ of constitutional laws^13^ in comparative studies needs to be reconsidered.^14^ With this cautionary note comes my second analytical point: what comparative constitutional studies need is method, not scientification. All the four varieties of comparative constitutional studies need to be ‘methodological’, as studying the constitutional phenomenon properly—comparatively or not—must have a well-framed research agenda.^15^ In developing its method, I argue, each comparative constitutional study should be guided by the interpretive needs entailed by its distinctive purpose instead of being driven by the pursuit of some assumed generalised scientific character in studying the constitutional phenomenon. In conclusion, I offer some critical reflections on the prevalent focus of comparative constitutional studies on master-text constitutions, case reporters and other formal institutional phenomena in constitutional ordering. With this ‘positivist’ propensity,^16^ I suggest, comparative constitutional studies may unwittingly prevent the emergence of rich studies of the constitutional phenomenon that dare to step out of the law-oriented positivist territory in constitutional scholarship.^17^ Apart from the critique of the current condition of the field, two contributions are expected of the identification of the four varieties of comparative constitutional studies and their distinct purposes. First, it will shed light on the pluralism and interpretive character of comparative constitutional studies. Second, it will do justice to comparative constitutional studies and help to put the comparative turn in constitutional scholarship in its place.

Besides the terminological definitions accompanying the relevant statements as the discussion proceeds, a clarification of key terms used in this article is warranted. By ‘the constitutional phenomenon’, I mean that which is *considered* of significance to the creation, operation, destruction and other aspects—directly observable and otherwise—of the project of constitutional ordering.^18^ As its significance is a result of consideration—and a collective one at that—it is embedded in a political community where its inhabitants interact with each other and with their environment, including other communities. It is in this sense that the constitutional phenomenon is also a ‘*social* phenomenon’, but is not absorbed into ‘the social’—the field of knowledge in the social sciences that is to be known chiefly through observation, discovery and causal explanation.^19^ Thus, ‘interpretation’ in constitutional studies, for present purposes, is distinct from ‘the interpretive turn’ in the ‘human sciences’ as it is not oriented towards the discovery of the intention of the constitutional subject under study, as Ian Shapiro suggests.^20^ Rather, interpretation here refers generally to the understanding—that is, the making sense of—the constitutional phenomenon by virtue of (comparative) constitutional studies, including through causal explanation.^21^ It is also worth noting that for present purposes, ‘knowledge’ is broadly understood to include the new perspectives and outlooks that help us to imagine an alternative world through seeing the familiar differently, alongside the epistemic progress engendered by ‘scientific’ methods.^22^

My argument proceeds in two stages. In the first, I classify comparative constitutional studies into the doctrinal, LS, documentary and cultural varieties, and critically discuss each in light of its distinct purpose in order. Following the critical reconstruction of the four varieties of comparative constitutional studies in terms of their interpretive needs, I take on the comparative turn in constitutional scholarship in general in the remainder of this article, with a focused examination of the myth of scientism in comparative constitutional studies. In conclusion, I summarise the argument and strike a cautionary note on the comparative turn in studying the constitutional phenomenon as it may inadvertently impoverish constitutional scholarship in general.

## 2. Seeking Illuminating Light in Foreign Oracles

Before ‘comparative constitutional studies’ displaces ‘comparative constitutional law’ as the orthodox designation of conducting constitutional research from comparative perspectives,^23^ constitutional comparison was essentially a lawyer business.^24^ This lawyer business does not disappear from the landscape of constitutional comparison.^25^ As with their predecessors, lawyers today, regardless of whether they are associated with academia or with legal practice, continue to be tasked to correctly interpret the law—including the *legal* constitution^26^—in order to faithfully carry out what the law mandates.^27^ To this lawyer business, what the late Justice Scalia of the Supreme Court of the United States called ‘a matter of interpretation’ speaks well.^28^ With constitutional issues boiling down to a matter of interpretation in the lawyer’s hand, the debate over methods of interpretation comes into focus in constitutional scholarship.^29^ It is as if finding the right method also guarantees the correct interpretation, setting the lawyer on the path towards the ‘right answer’.^30^ Against this backdrop arises the debate as to whether learning from fellow lawyers in other jurisdictions helps with correct interpretation of constitutional precepts.^31^

To those who value comparative constitutional law, constitutional interpretation can benefit from judicial opinions and legal commentaries of different jurisdictions.^32^ When they belong to the same ‘legal family’,^33^ such opinions and commentaries can be drawn upon for inspiration or adoption. When they come from a jurisdiction with a different legal tradition, there remains something to be learnt from the work of fellow lawyers. The shared common legal reasoning defines the identity of lawyers and helps judicial opinions and legal scholarship to transcend jurisdictional boundaries,^34^ facilitating the migration of constitutional ideas.^35^ As more and more constitutional principles and doctrines migrate across jurisdictions and are adopted by a wide range of constitutional orders, more often than not national apex courts and lawyers in general in different jurisdictions encounter the same principles and doctrines—a phenomenon which Jeremy Waldron calls ‘intertexuality’—in applying domestic constitutional law.^36^ Under such circumstances, judges and scholars are seen as virtually engaging in the interpretation of the same principles and doctrines—albeit differently formulated—in different fora at different times even if they are actually interpreting their own constitutions. Thus, how fellow lawyers in different jurisdictions have rendered the same principles or doctrines that come before the court (as well as the scholar’s study) can shed light on the latter’s understanding of such principles or doctrines in constitutional interpretation.^37^ This is why ‘judicial dialogue’ has come into prominence in comparative constitutional scholarship.^38^

Scepticism about the value of comparative constitutional law in constitutional interpretation is not uncommon and has been well documented.^39^ Cherry-picking is one of the most cited reasons by the naysayers of judicial dialogue and doctrinal comparison in general.^40^ Judges who draw on relevant decisions rendered by foreign or international courts, and scholars who look beyond the domestic constitutional landscape in search of answers to unresolved constitutional issues at hand, are accused of arbitrarily selecting foreign or international examples, if only to confirm their predetermined conclusion.^41^ To the naysayers, judicial *dialogue* is a misnomer. Throughout this ostensible deliberation among lawyers across jurisdictions, only the party who does the comparison and then decides the case (or resolves the issue at hand in the case of scholars) is active in this process. At the other end of such a purported judicial dialogue is the silent, passive source of reference that has no agency in this cross-jurisdiction learning process.^42^ Far from communication, such an exercise is akin to the practice of citation in scholarly writings.

These concerns are valid and have received serious responses from constitutional comparativists.^43^ Without repeating the argument in response, I suggest that, to assess the conduct of comparison in this regard, we need to be clear about the purpose of the doctrinal variety of comparative constitutional studies in the first place. In determining the purpose of doctrinal comparison, two values attached to the comparative approach to constitutional interpretation need picking out: knowledge and authority. As in other areas,^44^ asserting authority in constitutional interpretation—within and without the court—is closely intertwined with the knowledge about that which is subject to interpretation.^45^ The more solid the base of knowledge an interpretation rests on, the more authoritative an interpretation can claim to be. Thus, to enhance the authority of constitutional interpretation, all the analytical tools and methods that can buttress the epistemic basis of interpretation can be considered. From the relationship between authority and knowledge, it follows that we have no reason to categorically rule out the comparative approach—unless the comparative approach has proved *absolutely* detrimental to the development of constitutional knowledge.^46^

Yet, the asserted authority in constitutional interpretation does not *depend on* knowledge, even though the latter *may* contribute to the former. An interpretation of constitutional precepts that moves in the oppositive direction of the relevant knowledge remains authoritative in the sense that it commands compliance when it is rendered by a state organ with the mandate to settle the constitutional matter.^47^ In this kind of interpretative exercise, the foreign or international examples selected by the interpreting state organ are not necessarily meant to add epistemic value proper. Rather, those cited examples are instrumental to the rationalisation of the asserted authority in constitutional interpretation.^48^ Call this kind of constitutional comparison ‘jurispathic’—to borrow a term from the late Robert Cover’s legal lexicon.^49^ It should be noted that judges are not the only participants in jurispathic constitutional comparison. Academics who become advocates for domestic constitutional causes and pivot their doctrinal comparison to public causes they identify themselves with may prioritise interpretative authority through their advocacy over the enhancement of comparative knowledge in constitutional scholarship.^50^ Applied jurispathically, constitutional comparison—within and without the judicial forum—is not innocent of the naysayers’ accusation.

A different picture emerges when the focus is on the knowledge engendered by doctrinal comparison rather than the authority attributed to it. When detached from the authority to settle constitutional issues or to claim victory in public advocacy, constitutional comparison is then pivoted to finding answers to the unresolved constitutional matters that come to the interpreter’s attention.^51^ At such instances of interpreting, the purpose of the doctrinal variety of comparative constitutional studies is to seek illuminating light from sources beyond the domestic constitutional landscape to help to interpret constitutional norms correctly. Whether comparison is capable of shining the ‘jurisgenerative’ light sought by the interpreter remains a question to be fully answered.^52^ Yet, this question should not be conflated with that of the jurispathic (mis)application of the comparative method, which is driven by the assertion of authority.

Once the relationship between authority and knowledge is unpacked, the purpose of gaining better knowledge of how to interpret constitutional norms correctly comes to light and the value of constitutional comparison can be fairly appreciated. As discussed above, in reading constitutional master texts and case law, national apex courts and constitutional scholars in general under the condition of intertextuality can learn from the ‘oracles of the law’^53^ from afar who nonetheless share the lawyer’s way of reasoning.^54^ Whether this cross-jurisdiction learning will lead to a new *jus gentium*, as Waldron contends, remains to be seen.^55^ Whether doctrinal comparison should be aimed at the identification of some global standard in the interpretation of the constitutional concepts and principles that have spread worldwide is open to debate, too.^56^ What matters is that, from the lawyer’s perspective, correct interpretation continues to occupy the centre stage in reading the constitution. For the purpose of correct interpretation, doctrinal comparison is valuable.

Before leaving doctrinal comparison for the next variety of comparative constitutional studies, I should note that the risk of academic knowledge and authority being jurispathically used to buttress the judicial assertion of authority is real, given the close-knit relationship between the judiciary—the official authority holder—and academia—the assumed knowledge reservoir.^57^ Yet, such concerns should not be used as an excuse for discouraging learning through comparative studies. Instead, they should just serve as the reminder of what comparativists should be mindful of when doing doctrinal comparison.

## 3. Finding the Cause through ‘Scientific’ Explanation

Juxtaposed to doctrinal comparison, the LS variety of comparative constitutional studies seeks to better understand constitutional law in light of the broad social context. Although LS comparison continues with the Law and Society movement as a reaction to the doctrinal approach to legal studies in general and is increasingly aligned with the social sciences,^58^ its modern forebear is none other than Montesquieu.^59^ It is true that Montesquieu’s association of the spirit of the laws with external factors such as climate and geography shows the primitive nature of his nascent social scientific and comparative studies of constitutional ordering.^60^ Nevertheless, his attempt to make sense of the constitutional phenomenon in light of external societal factors sowed the seeds of the LS variety of comparative constitutional studies.^61^

As with other contextual approaches to legal studies rallying around the Law and Society banner, LS constitutional comparison has seen changes in its composition. Generally speaking, the Law and Society approaches arose in reaction to the formalistic character of doctrinal studies of law.^62^ Sociological jurisprudence,^63^ continental sociology of law,^64^ legal realism,^65^ the broad-church Law and Society movement^66^ and the later, more ideology-aligned, critical and economic analyses of law^67^ are all attempts to seek a better explanation of law than doctrinal studies. Situating law in the social context, such external approaches promise to enhance our understanding of it by taking extralegal factors into account. Notably, as the above successive contextual approaches suggest, the identity of extralegal studies of law has been continuously subject to contestation. Power, economic rationality, race, gender and institutions of political economy all compete to claim to hold the key to better legal knowledge—as if a genuine or, rather, scientific understanding of law depends on the external perspective from which to study it.^68^

Notwithstanding its grounding in the diverse Law and Society approaches, LS comparison has come under critical scrutiny when it is increasingly opened up to the social sciences. From the perspective of social science, the traditional LS comparison has suffered the same problem as its 18th-century ancestor aspiring to discover the spirit of the laws: without systematic methods, such external knowledge of comparative constitutional law is as lacking in analytical rigour, and thus as short of scientific quality, as doctrinal comparison.^69^ In response to such criticisms, social sciences-inspired LS comparativists treat constitutional ordering as a multifaceted social phenomenon. They look into constituents of constitutional ordering, such as institution, actor, norm, the social condition and, of course, the master-text constitution itself, and analyse how such constituents work in relation to the external context in which they are situated.^70^ Steeped in the Law and Society tradition, LS comparison has gradually evolved from general contextual approaches to law into a discipline of the social sciences.

As discussed above, doctrinal comparison has long been criticised for arbitrary selection of comparative examples. To be scientific, LS comparison needs more than just exploring the constitutional phenomenon from extralegal points of view that its forerunners had started.^71^ It needs to address the issue of arbitrary selection.^72^ One way to resolve this issue is to rid LS comparison of selection. When constitutional comparison is conducted on a global scale, there will be no selection, let alone arbitrary selection. Ran Hirschel’s pioneering work on the disparate constitutional status of city in the ‘new’ and ‘old’ constitutional worlds through a global survey of master-text constitutions informed by social scientific insights is evidence that LS comparison can go global.^73^

Yet, going global is not the only way to align LS comparison with scientific disciplines. As with other comparative studies in the social sciences, LS comparison can choose to focus on some constitutional institutions, say, political parties, of selected countries to generate comparative insights. Regardless of the scope of comparative reach, the new strain of LS comparison owes its scientific quality to methods of analysis widely employed in the social sciences, such as interview, focus group study and statistical modelling.^74^ For example, to see how political parties influence participatory constitution making, a focused systematic comparison of the Brazilian and South African experiences in their constituent moments has its own scientific value.^75^

It should be noted that traditional LS comparison does not disappear from the landscape of comparative constitutional studies amid the continuing rise of the social sciences in this field. After all, social scientists are not freed of questions of whether and to what extent various aspects of society, or, rather, ‘the social’—the context in which LS comparison is situated—can be fully grasped by their research tools.^76^ Despite their variance, the traditional and scientific strains of LS comparison are united in purpose. Departing from doctrinal approaches, LS comparison in both strains is aimed at understanding constitutional ordering as a phenomenon embedded in ‘the social’.^77^ LS comparativists thus look to factors external to the object of study—regardless of whether it is the master-text constitution itself or other observable constitutional phenomena—to explain what engenders the object and how the object becomes what it is.^78^ In making sense of constitutional phenomena in different jurisdictions, the LS variety of comparative constitutional studies in general is expected to bring forth what enables their current condition by looking beyond doctrine. The whole picture of the constitutional phenomenon as part of ‘the social’ is the common pursuit of the traditional and scientific strains of LS comparison, although they are not in unison when it comes to what the best depiction is.

To get a better sense of the purpose of LS comparison, it helps to point out that the doctrinal variety of comparative constitutional studies is no less concerned with what enables the current condition of the constitutional phenomenon under examination than its LS counterpart. Doctrinal comparison turns to constitutional precepts to clarify the normative underpinnings of the current condition,^79^ while LS comparison interprets the current condition of the constitutional phenomenon in a unique way—in light of the data generated by social scientific research tools or the empirical findings otherwise made. On the one hand, LS comparativists do not brush aside the importance of the doctrinal interpretation of constitutional master texts or the implicit constitutional principles to the phenomenon they try to make sense of.^80^ On the other hand, as will be further discussed next, they are also aligned with their counterparts in documentary comparison in a way: both are dissatisfied with traditional comparative constitutional law handicapped by methodological amateurism, and take interest in the influence of constitutional design choices of individual Constitutions on the social constitutional phenomenon. Nevertheless, LS comparativists part company with their fellow constitutional comparativists because the phenomenon they try to make sense of does not receive a satisfactory explanation or answer from doctrinalists or documentarians. To them, neither doctrine nor constitutional master text alone is enough.^81^

For example, from the LS point of view, to answer the question whether secession is a right inhering in constitutional governance, the answer would fall short if it simply relies on how individual national apex courts have derived it from their constitutional master texts or how many Constitutions in the world have provided for it.^82^ LS comparativists would situate the question in the context in which it is formulated—to what issues a constitutional right to secede is expected to respond. They would further address the question of the right to secession in terms of its effectiveness in response to the underlying issues and its further ramifications. Further powered by the social sciences, they can even rest such assessments on empirical evidence.^83^ By situating the constitutional phenomenon under examination in the external context, LS comparison in general focuses on the causes of its current condition, while social scientific tools further enable LS comparativists to account for the object of comparison contextually and scientifically through systematic explanation of its extralegal causes.

Taken together, in comparative constitutional studies, the matter of LS interpretation is more explanation of constitutional ordering than interpretation of constitutional instruments or their derived doctrines. The purpose of comparative constitutional studies in the LS variety is to better identify the causes of the constitutional phenomenon concerned by means of rigorous research and therefore to strengthen or alter current relevant explanatory theories concerning constitutional ordering.^84^ In sum, the interpretation LS comparison gives of the social constitutional phenomenon eventually takes on the character of scientific explanation following its social scientific turn.^85^

## 4. Discovering the ‘Law’ of the Effective Constitution

The designation of documentary comparison indicates the defining feature of this variety of comparative constitutional studies: the centrality of the ‘C-documents’—such as the large-C Constitution or the master-text constitution—to constitutional comparison.^86^ It is a recent addition to the repertoire of comparative constitutional studies, which stands in contrast to the long tradition of doctrinal and early LS comparison. Apart from the generation gap, the documentary variety has a complicated relationship with the other two. Consider first its relationship with doctrinal comparison. While documentarians and doctrinalists share interest in constitutional instruments, their shared interest stops at the C-documents. For documentarians, the large-C Constitution does not just constitute part of their interpretation. It further occupies their attention.^87^ From the effectiveness of constitutional bills of rights^88^ to the right to resist,^89^ from global constitutional values^90^ to the life span of individual Constitutions,^91^ and from social rights^92^ to the influence of American constitutionalism,^93^ documentarians take on a wide range of constitutional subjects and issues by comparing individual large-C Constitutions around the globe. Those C-documents are where documentarians find their answers to questions such as whether there exist globally shared constitutional norms,^94^ what purpose a constitutional right to resist serves^95^ and whether a country’s compliance in one category of constitutional rights is indicative of its constitutional compliance in others.^96^ In a nutshell, constitutional master texts, to doctrinalists, indicate what concepts and principles have been commonly adopted,^97^ whereas in the hands of documentarians, the C-documents speak for themselves—to be more precise, they are made to speak with the aid of scientific methods.

Let us take a closer look at how documentarians make their object of comparison—the large-C Constitutions—speak before exploring the relationship between LS and documentary comparison. In contrast to doctrinalists turning to case law compendia or academic writings for guidance in reading master-text constitutions, documentarians seek illuminating light from statistical methods such as regression analysis.^98^ To decipher the C-documents, they further read the results generated by such methods against external indices created by third parties such as the World Bank’s World Development Indicators^99^ or Freedom House’s annual civil liberties scores.^100^ Ultimately, documentary comparison as a scientific approach to comparative constitutional studies is not so much about reading and interpreting as about documenting and decoding the large-C Constitutions.^101^

Documentary comparison’s self-image of being scientific and methodological as manifested in its distinctive approach to ‘reading’ constitutional master texts is also revealing of its zeitgeist. To make comparative constitutional studies scientific, documentary comparison tries to steer clear of the features characteristic of both doctrinal and LS varieties that are suspected of subjective bias. As noted above, arbitrary selection has been one of the issues haunting doctrinal comparison. Although the bias problem eases up for LS comparativists thanks to the employment of social scientific analysis tools, documentarians are generally disaffected by comparison that requires selection. In the documentarian’s eye, selection is inescapably subjective, contradicting the pursuit of objectivity in science. ‘Be global’ thus becomes the *de facto* motto of documentary comparison as global survey apparently saves documentarians from the woes of selection.^102^ Notably, the global reach characteristic of documentary comparison is only made possible by external conditions, including scientific progress. On the one hand, driven by the wave of globalisation, large-C Constitutions of individual countries have been made accessible to international academia with their translation into English.^103^ On the other hand, the advances in word processing and other text comparison software enables efficient comprehensive comparison of numerous constitutional master texts—historical and contemporary—across the globe.^104^ Without this scientific progress, documenting and decoding at the core of documentary comparison would be too onerous to have the global reach we see today. Thanks to the emergence of English as the lingua franca of the C-documents in comparative materials and the progress in document software, documentarians are able to document and code constitutional master texts on a global scale.

The scope of comparison is not the only feature that characterises documentary comparison as a new variety of comparative constitutional studies. As noted above, despite their variance in the weight doctrinal interpretation deserves, both LS comparativists and doctrinalists pay attention to how such interpretation bears on the constitutional phenomenon they try to make sense of. They acknowledge that constitutional master texts alone do not give their readers the whole constitutional picture. In contrast, to documentarians, constitutional master texts are the foremost, if not exclusive, raw materials of comparative studies.^105^ For documentarians, looking beyond the C-documents risks muddying the object of study and comparison.^106^ Documentary comparison instead centres on large-C Constitutions. Relatedly, they stand apart from their fellow constitutional comparativists on methodology. Traditional skills of legal interpretation and analysis underpinning doctrinal interpretation are found insufficient to produce scientific comparative constitutional studies.^107^ Here starts the kinship between documentary comparison and the scientific strain of LS comparison.

Notably, as suggested above, not all the varieties of comparative studies discussed so far are mutually exclusive in terms of object of analysis and method. The relationship between LS and documentary comparison is such an example of non-exclusivity. Documentary approaches overlap with LS approaches in causal origins and consequences.^108^ Echoing the social scientists in the LS variety, documentarians contend that to be scientific, comparative constitutional studies need the aid of new research skills underpinned by robust methods of analysis. Coding is just the first step towards making sense of constitutional master texts around the globe. Statistical analysis and other empirical methods are needed to reveal the message implicit in the data extracted from constitutional master texts that is not accessible to those who only read the documents with their eyes and minds. Regression analysis illustrates how the law—the lawlike regularity—immanent in constitutional master texts can elude doctrinal analysis, only to be deciphered *scientifically*.^109^ Emphasis on scientific rigour and methodological sophistication in documentary comparison gives away the influence of the social sciences. Put bluntly, documentary comparison is a sibling of the scientific strain of LS comparison that has been empowered by the social sciences.^110^ Despite the family resemblance, documentary comparison is nonetheless distinct from social sciences-powered LS comparison for its choice of constitutional phenomenon to explore: the large-C Constitutions. The purpose of documentary comparison explains its unique focus.

Notably, both LS comparativists and documentarians are interested in the effectiveness, functions or implications of constitutional design choices. Thus, individual blueprints of constitutional design as presented in the C-documents must be consulted in the first place.^111^ To LS comparativists, factors external to the object of study—ie the environment the blueprint of constitutional design finds itself in—must then be taken into account. Yet, for documentarians, external factors only compromise the mission because their task is to bring forth factors ‘endogenous’ to constitutional design itself.^112^ To this end, they code the large-C Constitutions and subject the coded data to methodologically rigorous analysis to discern any ‘trends’, ‘patterns’ or what Ran Hirschl calls ‘general laws’ immanent in the design.^113^ In making an empirical appraisal of whether and, if so, how constitutional design influences society,^114^ they further read the results derived from coding and empirical analysis against the external indices of the relevant social phenomena, such as human rights performance, economic prosperity or average life expectancy.^115^ Notably, following the identification of trends or patterns derived from the coding and empirical analysis of constitutional master texts, such results are subject to evaluation, which is a function of interpreting.^116^ Only after interpretation and evaluation can the effectiveness of constitutional design be appraised.^117^ Yet, to find the effective constitutional choices in enhancement of human rights, living standards or longevity, for example, it turns out that interpretation and evaluation in the documentary variety virtually rely on external indices created by third parties. Seen in this light, documentary and LS comparison (as in the case of the latter’s scientific strain) ultimately diverge: the focus of documentary variety is on the factors endogenous to the object of study—constitutional design choices and their immanent pattern or trend to be extracted from the C-documents—while social sciences-powered LS comparison looks beyond the object itself.^118^

The empirical appraisal of the effectiveness of constitutional design through scientific and methodological comparison of individual large-C Constitutions is not without question.^119^ For present purposes, suffice it to say, documentary comparison cannot do without interpretation, even if its focus is on the blueprint of constitutional design—the large-C Constitution—and its real-world effects. By focusing on the master-text constitution itself and ‘reading’ it under the instruction of statistics instead of legal hermeneutics, documentary comparison aims to exclude the influence of the document reader’s bias in comparative constitutional studies. To maintain the scientific character of comparative constitutional studies, documentarians even move ahead of their fellow social sciences-powered LS comparativists. They interpret their findings in light of the external indices attributed to seemingly universal international organisations such as the World Bank and the United Nations or esteemed non-governmental institutions like Freedom House and Amnesty International^120^ in the hope of adding another layer of objectivity to their comparative analysis.^121^ In documentary comparison, it is the systematic lawlike features of various trends, patterns or regularities identified in large-C Constitutions that are to be interpreted and evaluated in the light of third-party criteria.^122^ In sum, documentary comparison remains a matter of interpretation and cannot get away from the issues that have bothered every exercise of interpretation, including the influence of bias.^123^

## 5. *Reading the Constitutional* Nomos

Rooted in the tradition of humanities,^124^ cultural comparison occupies a unique place in the landscape of comparative constitutional studies. On the one hand, to cultural comparativists, reading and interpreting a constitution is more than an exercise of doctrinal analysis and legal reasoning. The meaning of each constitutional order is to be gleaned from the relationships of social life in the political community that knit together the relevant ‘webs of significance’,^125^ while such webs of significance both frame and reflect the way the constitutional order and its effect on social relationships are grasped.^126^ On the other hand, aimed at interpretation, cultural comparison does not seek to explain the constitutional phenomenon in causal terms. Culturalists set themselves apart from social scientists in both the LS and documentary varieties of comparative constitutional studies, as they try to make sense of constitutional ordering through hermeneutic engagement instead of social scientific analysis. In sum, the cultural variety approaches comparative constitutional studies as a matter of interpretation in the tradition of humanities.^127^

Being a branch of the humanities, cultural comparison reads constitutional ordering as a manifestation of the broad culture of the relevant jurisdiction. Through the lens of the cultural study of law,^128^ the constitutional phenomenon is viewed as part of the world of meaning that gives identity to both the society and those inhabiting it, bringing forth a shared normative universe, ie a *nomos*.^129^ The *nomos* not only structures the time and space of a constitutional order, but also makes the constitutional subject under that constitutional order.^130^ From the perspective of cultural comparison, studying the constitution is a matter of interpretation whose object is the constitutional *nomos*.^131^ Thus, in the culturalist’s eye, the purpose of comparative constitutional studies is to make sense of the cultural significance of constitutional ordering in different jurisdictions by reading individual constitutional *nomoi* in their own terms.^132^ Through comparison, culturalists try to understand the meaning of each constitutional order by reading it in terms of its own webs of significance and what Charles Taylor calls ‘social imaginary’.^133^

Despite its purpose and uniqueness, cultural comparison is not insular in the universe of comparative constitutional studies. To grasp the cultural meaning of the Constitution, cultural comparativists need to understand what it provides for and how judges have interpreted constitutional provisions. Results of doctrinal comparison are ingredients for culturalists. Moreover, as noted above, culturalists identify the meaning of the constitutional phenomenon in webs of significance and read constitutional orders in light of individual constitutional imaginaries. Anthropological studies—part of the social sciences in the modern division of academic disciplines^134^—and other Law and Society approaches point culturalists to the web of significance in which the meaning of the constitutional phenomenon is to be found.^135^ Thus, cultural comparison inevitably engages with philosophy and social theories in bringing constitutional imaginary to bear on the interpretation of constitutional orders.^136^ Seen in this light, cultural and LS comparison are not mutually exclusive. Even documentary comparison can be beneficial to culturalists. When drawn into the interpretation of the lawlike patterns and trends identified by documentarians, culturalists interrogate allusions of the transnational normative universe emanating from such global constitutional trends.^137^ Taken together, cultural comparison is an engagement with constitutional orders under an overarching framework of interpretation that is to be found anthropologically or philosophically. It bears emphasis that it is the engagement itself—not the framework informing the engagement—that constitutes cultural interpretation proper and the focus of cultural comparison discussed here.

With the approach and purpose of cultural comparison and its relationships with other varieties of comparative constitutional studies brought to light, its characteristic features are also revealed. First, situating constitutional orders in the web of significance, culturalists consider the meaning of constitutional master texts, doctrines, institutions and other constitutional phenomena in relation to that which is exogenous to such phenomena. To this extent, cultural comparison parallels its LS counterpart: to both of them, the external environment the master-text constitution and constitutional law find themselves in is that which matters in making sense of a constitutional order. Yet, they tend to diverge on what constitutes the external environment and how to get to it. As discussed above, to the LS variety, the external environment is societal in nature—‘the social’—and it takes both explanatory theory and research methods developed outside traditional legal scholarship—ie in the social sciences—to get to it,^138^ although LS comparativists may draw on accounts of power that link to cultural approaches.^139^ In contrast, cultural comparison looks to the humanities for help since what it needs to get to is not so much society as that which gives meaning to society and various social phenomena.^140^ In a nutshell, the *nomos* is what cultural comparison tries to approach in bringing forth the meaning of the constitutional phenomenon. And this brings up the second feature of cultural comparison.

To read a constitutional *nomos* deeply in order to understand the cultural significance of the constitutional phenomenon suggests that cultural comparison resists the global survey that predominates in documentary comparison and is present in some of the scientific strains of LS comparison. Consider the fox–hedgehog parable in Isaiah Berlin’s philosophical world.^141^ Students of the constitution can focus on broad survey in the world of comparative constitutional studies as if to follow the fox-type philosopher to know as many things as possible. Or, they can choose to act like a philosophical hedgehog to focus on one big thing through deep engagement.^142^ To those who consider the constitutional phenomenon from the cultural perspective, the choice is clear: focusing on a single constitutional *nomos* and reading it carefully and deeply—just like the hedgehog—holds the key to making sense of the constitutional phenomenon.^143^ Cultural comparison is thus leaning towards single case studies.^144^

The dominance of single case studies is not only a result of learning capacity as suggested above, but also an expression of the culturalist’s attitude towards comparative constitutional studies in general. As suggested above, not only documentary and LS comparison, but also doctrinal comparison is pivoted to some overall framework across jurisdictions and societies in studying constitutions, whether it is some veiled common standard, well-received explanatory theory or shared normative value. In contrast, cultural comparison is singular in character. It is oriented towards thick description of the constitutional phenomenon embedded in webs of significance in light of its own constitutional imaginary.^145^ This singular character gives away another characteristic feature of cultural comparison.

As noted above, cultural comparison turns to the humanities in interpreting the constitutional phenomenon. Even so, just as with their fellow comparativists in other varieties of constitutional comparison, cultural comparativists also read constitutional master texts and case reporters while taking into account institutions, norms and other constitutional phenomena. What distinguishes cultural comparison from other varieties of comparative constitutional studies is that its ‘reading’ list extends further to novels, parables, films and other narrative expressions that tell ‘stories of peoplehood’ and illuminate constitutional imaginaries.^146^ To grasp the cultural significance of constitutional ordering, cultural comparison rejects determining the scope of studies and defining the interpretation based on the observable facets of the constitutional phenomenon such as doctrine, text, institution, actor or event. Even a play can shed illuminating light on the relationship between death, revenge and justice—a complexity the constitutional *nomos* has to grapple with, with or without the codification of human dignity in the Constitution.^147^

To complete the discussion of cultural comparison, one more question needs to be answered. If culturalists have the predilection for deep reading with a focus on single case studies, can such work still be considered comparative? The answer lies in our appreciation of the works of cultural comparison. Culturalists have joined forces to present volumes or symposia on focused themes such as justice, legitimacy or sovereignty through cultural studies of individual constitutional orders.^148^ For purposes of cultural comparison, constitutional taxonomy of this kind still matters, even though doubts have been cast on this genre as comparative constitutional law continues to evolve into (scientific) comparative constitutional studies.^149^ Also, works of cultural studies of constitutional law focused on limited—say, two—jurisdictions as case studies should not be treated as less valuable than comparative constitutional studies that are noted for a ‘fair’—or, rather, large—number of cases in comparison. In a way, single case studies are to global surveys in constitutional comparison as area studies are to international relations in political science.^150^ Just as both area studies and international relations are essential to understanding international politics, so single case studies and global surveys together contribute to comparative studies of the constitutional phenomenon. Moreover, as cultural comparison makes sense of constitutional orders through the lens of constitutional imaginary, the study of different constitutional imaginaries in different political cultures is comparative in every aspect.^151^

## 6. *Constitutional Comparison in the Shadow of Scientism*

The multiple varieties of constitutional comparison are evidence of the thriving of comparative constitutional studies. With each variety having its own distinct purpose, comparative constitutional studies are far from a unitary scholarly enterprise. Guided by purpose, each variety of comparative constitutional studies seeks to make sense of the constitutional phenomenon through its defined object and scope of study. Furthermore, to serve its purpose, each variety needs to design the research properly when engaging with its identified object of study within the stated scope. Taken together, purpose not only frames the object and scope of study, but also helps to set the research agenda and choose the attendant method for comparative constitutional studies. Whether individual comparative constitutional studies live up to the standard of scholarship should be assessed in terms of the purpose-guided research design and the method adopted besides the argument and analysis. A comparative study of the constitutional phenomenon that fails the standard informed by the purpose of its variety is unsound. There is just no one-size-fits-all approach to constitutional comparison.

Yet, the diverse landscape of comparative constitutional studies is now under pressure amid the drive for more sophisticated and ‘scientific’ constitutional comparison in scholarship, despite the exhortation for ‘methodological pluralism’ and ‘analytical eclecticism’ in the field.^152^ As the trajectory of the four varieties of comparative constitutional studies suggests, LS and documentary comparison have emerged as efforts to address the contemporary unsatisfactory state of comparative constitutional law, while culturalists have long read and interpreted the constitution—alongside doctrinalists—in the tradition of humanities. Call the LS and documentary varieties the ‘reformation’ in comparative constitutional studies.^153^ The quest for reform or progress is characteristic of all disciplines in academia and has been the driver for the advancement of human knowledge—the epitome of which is the Scientific Revolution.^154^ Thus, the reformation of comparative constitutional studies driven by the LS and documentary varieties should come as no surprise. Yet, in pushing for reform, LS comparativists and their more recent fellow travellers powered by social scientific tools are disaffected by doctrinal comparison and comparative constitutional studies informed by the humanities in general.^155^ The scientific strain of LS comparison and its documentary counterpart stand out for their social sciences-framed method and research design. They do not enter the landscape of comparative constitutional studies so much for the reinforcement of other varieties as for the latter’s replacement.

Reminiscent of the linear, progressive outlook of the Scientific Revolution, the social sciences-powered reformation sees comparative constitutional law in the same way as modern sciences did ‘natural philosophy’ in the 17th century.^156^ In the eye of social sciences-inspired LS comparativists and documentarians, doctrinal and cultural varieties of constitutional comparison become regrettable old habits in comparative constitutional studies, evoking how natural philosophy went down with the nascent natural sciences in the modern age.^157^ The diversity of constitutional comparison speaks to such vestiges instead of something deserving celebration. Situated in the trajectory of the development of constitutional comparison, the diverse landscape of comparative constitutional studies now appears to be a transient scene before the culmination of reformation whereby comparative constitutional studies are expected to turn into a ‘scientific’ discipline. Beneath the surface of the landscape of diverse constitutional comparison runs an undercurrent of ‘social scientification’,^158^ raising issues concerning both the identity and health of comparative constitutional studies.

As noted above, the latent ‘social scientification’ of comparative constitutional studies is reminiscent of the development of natural sciences. Yet, the field of comparative constitutional studies is not quite in line with natural sciences. While natural sciences are aimed at ‘the discovery of new objects, phenomena, mechanisms, cures, technologies, and even theories’,^159^ comparative constitutional studies in each of the four varieties cannot dispense with interpretation. Natural sciences advance knowledge by empirically observing the world and discovering the real cause of an observable natural phenomenon or a new phenomenon itself—first through the naked eye and now mostly with the aid of scientific instruments, including artificial intelligence^160^—while purpose guides interpretation in comparative constitutional studies. There is no final definitive interpretation when it comes to the constitutional phenomenon. Framing and resulting from human interaction at the same time, constitutional ordering is not just an analysis of directly observable experiences of individual constitutional systems through the relevant data and findings.^161^ Rather, its significance is always circumstantial.^162^ It is in the continuing flow of interpretation that the constitutional phenomenon is continually illuminated and better grasped.^163^ Knowledge of comparative constitutional studies grows with interpretation of the constitutional phenomenon multiplying. This is no exception to the social scientists-comparativists in LS and documentary comparison who claim to interpret the constitutional phenomenon in the form of scientific explanation.^164^

Notably, unlike (natural) scientists who discover an unknown object through empirical exploration or design their experiment to discover the cause of the yet-to-be-explained phenomenon, the social scientists-comparativists in comparative constitutional studies need to define their object of observation in the first place.^165^ Their social scientific explanation of the constitutional question is attached to the defined object. Yet, the definition of a constitutional object to be explained in and of itself is an exercise of interpretation in the non-social scientific sense.^166^ This is no surprise as every constitutional phenomenon is overdetermined and embedded in a complex social milieu. There is no single cause *of* it.^167^ Leaving the constitutional phenomenon undefined, the social scientist-comparativist will be drawn into the problem of the infinite chain of causation.^168^ Thus, as in other social sciences, explanations offered by social scientists-comparativists in constitutional studies are always bounded—by the defined object.^169^ Only to that extent is the explanation of the constitutional phenomenon—or, rather, one facet of it—that results from comparison sound.

I should make it clear that nothing said above suggests that the social sciences have no place in comparative constitutional studies. On the contrary, social sciences-informed comparative studies have advanced our knowledge of the complex constitutional phenomenon tremendously and are welcome additions to comparative constitutional studies. What I have said of the social scientification of comparative constitutional studies is meant to emphasise that we should bear in mind that constitutional comparison comprises multiple matters of interpretation—each of which is guided by its distinct purpose. Social sciences-steered comparative studies are valuable when the purpose of constitutional comparison is to explain, say, the relationship between the change in the procedural rules regarding standing in judicial review and the professional status of lawyers who specialise in constitutional litigation *vis-à-vis* their peers in the legal practice.^170^ Yet, when the purpose is to understand the significance of such changes to the popular image of the constitution, the social sciences can still help but will have no claim to superiority as such understanding is more than just discovery and explanation. The popular image of the constitution can be mapped through public opinion polling, discourse analysis, interview of parties to judicial proceedings or dissection of how constitutional cases are narrated.^171^ Yet, which result best represents the popular image is beyond the control of the social sciences. In sum, for all the trajectory of the development of comparative constitutional studies as a whole, social scientification is far from the destiny of comparative constitutional studies. Instead of the golden standard, it should be treated as a sombre reminder of the importance of pluralism of comparative approaches in making sense of the constitutional phenomenon.^172^

Apart from issues concerning the identity of comparative constitutional studies as part of the research of social phenomena *vis-à-vis* natural sciences, the ostensible social scientification in constitutional comparison poses an indirect threat to the health of comparative constitutional studies as a discipline. As suggested above, the emergence of social sciences-steered constitutional comparison is seen as the epitome of intellectual sophistication in comparative constitutional law for its perceived objectivity, exhibiting a liking for scientism in the latent social scientification of comparative constitutional studies.^173^ To be scientific this way, comparative constitutional studies are expected to be objective in the sense of bias being excluded. ‘De-subjectivisation’—or, rather, ‘depersonalisation’—distinguishes scientific from unscientific comparative constitutional studies.^174^ On this view, the social sciences-informed LS and documentary varieties stand for scientific comparison; doctrinal and cultural comparisons fall under the unscientific category. As a result, *universal* features, *third-party* indices, *comprehensive* survey and ‘*large-N*’ methods, for example, are preferred to interpretation and single case studies in constitutional comparison. Is this a problem?

My answer is ‘It depends’. If the objective of study is to find out some lawlike regularities in, say, the formulation of freedom of speech and its limitation in written constitutions, the preference for social sciences-steered comparative studies is not unreasonable. Even so, constitutional comparativists should be clear that what an individual instance of constitutional comparison makes of the complex constitutional phenomenon is a result of epistemic construct.^175^ In the foregoing example, the findings of the enshrinement of freedom of speech in written constitutions remain imbued with values, despite the social sciences-informed sophisticated designed comparison, revealing the interpretive character of such comparative exercises. On the other hand, if deep understanding of the meaning of individual constitutional orders in their own cultural terms is the purpose of comparison, interpretation should be chosen over social scientific explanation, while global survey may well have to yield to single case studies.^176^ In a nutshell, pursuing (social) scientification as an end in and of itself can indeed make some varieties of constitutional comparison more sophisticated, but may inadvertently impoverish comparative constitutional studies as a whole.

The social sciences have enriched constitutional comparison by bringing new perspectives and research tools into comparative constitutional studies. Even more so, the emphasis the social scientist-comparativist places on research design and method is invaluable to every student of comparative constitutional studies,^177^ regardless of whether she is trained as a lawyer or approaches the constitutional phenomenon from the tradition of humanities. Although, as part of interpretation, comparative constitutional studies—in all the four varieties—cannot be bias-free,^178^ this does not license comparativists to turn interpretation into personal conviction, if only to manipulate.^179^ Interpretation—or, rather, how to do interpretation—is embedded in an interpretive community and needs to be guided by the purpose each exercise of interpretation aims to serve.^180^ Being methodological in the sense of having a well-framed research agenda should be the goal of all four varieties of comparative constitutional studies; becoming methodological without succumbing to (social) scientification makes comparative constitutional studies ‘scientific’ in the proper sense.^181^ All comparativists can learn from social scientists in framing the research agenda, but they should be cautious not to fall into the trap of what Ian Shapiro has said of the ‘method-driveness’ in the human sciences.^182^ While social scientification does not serve the purpose of comparative constitutional studies as a whole, the social sciences have indeed enriched our comparative understanding of the constitutional phenomenon. Seeing through the myth of scientism helps to do comparative constitutional studies properly.

## 7. Conclusion

Today, constitutional law is no longer the unconquered frontier in law for comparative studies, as Twining suggested over a decade ago. Comparative constitutional law is not only thriving, but also evolving into comparative constitutional studies that transcend the traditional field of legal scholarship. Ten years after the renaissance of comparative constitutional law was declared, I have taken a close look at the state of comparative constitutional studies in this article.

Starting with a survey of constitutional comparison, I suggested that comparative constitutional studies are far from a unitary scholarly enterprise. Rather, four varieties coexist in the diverse landscape of comparative constitutional studies. I discussed each of the four varieties of constitutional comparison—doctrinal, LS, documentary and cultural—in order, and argued that all of them are concerned with interpretation, despite their distinct purposes. In comparative constitutional studies, we see four matters of interpretation.

Although some varieties, such as the LS and the documentary, have emerged in response to the unsatisfactory condition of constitutional comparison associated with other varieties, I have tried to show that every variety of comparative constitutional studies has its own merits and limits. Thus, there does not exist a rank order among the four varieties. Instead, I stressed that purpose matters and it determines how comparative studies can be done properly. To achieve the ultimate goal of comparative constitutional studies—to help students of the constitution to make sense of the constitutional phenomenon in its various manifestations through understandings of different constitutional orders—the comparative approach needs to be employed discriminatingly.

Yet, as the developmental trajectory of current comparative constitutional studies suggests, the social sciences-informed additions to the comparativist repertoire—ie the scientific strain of LS comparison and the documentary variety—are now seen as heralding a new era of comparative constitutional studies, marked by its intellectual maturity and methodological sophistication. This reflects a quest for scientism in comparative constitutional studies. I took a critical look at the latent drive for social scientification in comparative constitutional studies and argued that pursuing (social) scientification regardless could inadvertently impoverish comparative constitutional studies as a whole. Falling under the spell of scientism, comparative constitutional studies would allow the tail of scientific methodology to wag the dog of purpose-guided interpretation in comparative understanding of the constitutional phenomenon. Failing to see the indelible interpretive element in constitutional comparison, comparative constitutional studies can still continue to thrive quantitatively, but exemplary and good comparative studies of the constitutional phenomenon may well be few and far between. The first step towards constitutional comparison is to see comparative constitutional studies as part of interpretation in continuing efforts to understand the multifaceted constitutional phenomenon and not to pursue (social) scientification at the expense of purpose.

As I noted at the beginning, comparative constitutional studies are valuable for contributing to good studies of constitutional ordering. The value of constitutional comparison would be thrown into doubt if it hardly improves our understanding of the multifaceted constitutional phenomenon and thus fails the standard of good studies. To do justice to the merits and limits of comparative constitutional studies requires a separate and full investigation. Nevertheless, it is worth asking here whether constitutional scholars may unintentionally narrow the scope of study and make only partial observation of the constitutional phenomenon itself in enthusiastically pursuing comparative studies, even though the geographical scope of such a pursuit expands. As noted above, constitutional ordering is a multifaceted phenomenon. Apart from observable aspects of constitutional ordering, such as institution, procedure, doctrine, convention and, of course, the master-text constitution, along with other statues of constitutional significance, each constitutional order has other factors that contribute to its functioning.^183^ Stopping at observing the constitutional phenomenon as manifested in norms, procedures and other formal institutions without digging into those factors that are not directly visible produces only a partial account of the constitutional phenomenon. And this is why we should be cautious when it comes to comparative constitutional studies. As master-text constitutions, case reporters and other, more accessible materials represented in legal instruments occupy the focus of attention in constitutional comparison,^184^ comparative constitutional studies reveal a positivist propensity. Leaving this propensity unaddressed and modelling constitutional studies after the comparative trend may unwittingly give undue weight to the constitution’s law elements and other formal factors at the cost of the less perceptible facets of the constitutional phenomenon.^185^ It is the making sense of the constitutional phenomenon properly, instead of pursuing comparison in and of itself, that should guide studies of constitutional ordering. When taking the comparative path in the field of constitutional studies, we should always stay alive to the positivist trap in constitutional comparison.^186^

The rise of comparative constitutional studies is part of the never-ending effort to make sense of the multifaceted constitutional phenomenon. To turn the renaissance of comparative constitutional law into the golden era of constitutional studies, we should take the comparative approach seriously and employ it in a way that the purpose of comparison can be well served. Failing to see the promise of comparative constitutional studies and their limits at once, attempts to make sense of the constitutional phenomenon *comparatively and properly* will be futile.

## Footnotes

1 Twining’s lectures were later published as William Twining, *Globalisation and Legal Scholarship* (Wolf Legal Publishers 2011).

2 ibid 20–8.

3 ibid 21.

4 Ran Hirschl, *Comparative Matters: The Renaissance of Comparative Constitutional Law* (OUP 2014). Notably, Ran Hirschl traced the roots of the renaissance of comparative constitutional law to the mid-1980s (151). For a different narrative of the ‘renaissance of comparative constitutionalism’, see AE Dick Howard, ‘A Traveler from an Antique Land: The Modern Renaissance of Comparative Constitutionalism’ (2009) 50 Va J Int’l L 3, 5–14.

5 See Silvia Suteu, ‘The View from Nowhere in Constitutional Theory: A Methodological Inquiry’ in Dimitrios Kyritsis and Stuart Lakin (eds), *The Methodology of Constitutional Theory* (Hart Publishing 2022) 341. The volume of research handbooks recently published on comparative constitutional law is evidence to the comparative turn. Examples include Tom Ginsburg and Rosalind Dixon (eds), *Comparative Constitutional Law* (Edward Elgar 2011); Michel Rosenfeld and András Sajó (eds), *The Oxford Handbook of Comparative Constitutional Law* (OUP 2012); Roger Masterman and Robert Schütze (eds), *The Cambridge Companion to Comparative Constitutional Law* (CUP 2019); Xenophon Contiades and Alkmene Fotiadou (eds), *Routledge Handbook of Comparative Constitutional Change* (Routledge 2020); see also Gary Jacobsohn and Miguel Schor (eds), *Comparative Constitutional Theory* (Edward Elgar 2018); cf András Jakab, Arthur Dyevre and Giulio Itzcovich (eds), *Comparative Constitutional Reasoning* (CUP 2018). The United States remains an outlier in this development. Compare Mark Tushnet, ‘The Boundaries of Comparative Law’ (2017) 13 EuConst 13, 14, with Michel Rosenfeld, ‘Comparative Constitutional Analysis in United States Adjudication and Scholarship’ in Rosenfeld and Sajó (idem) 38, 39.

6 Such questions had been raised in relation to comparative law and comparative politics well before the declared renaissance of comparative constitutional law. See Jennifer Widner, ‘Comparative Politics and Comparative Law’ (1998) 46 Am J Comp L 739.

7 See René van Woudenberg, *The Epistemology of Reading and Interpretation* (CUP 2021) 180.

8 For present purposes, ‘comparative constitutional studies’ is understood as the conduct of constitutional research from a comparative perspective and the resulting scholarship. Thus, comparative constitutional law is not contrasted to, but part of, comparative constitutional studies. My present concern is with comparative constitutional studies as understood above, not comparative constitutional law proper. For comparative constitutional law as defined in contrast to interdisciplinary comparative constitutional studies, see Hirschl, *Comparative Matters* (n 4) 163–91.

9 Hirschl uses the term ‘explanation’ instead. ibid 226. See also NW Barber, *The Constitutional State* (OUP 2010) 1–5.

10 Ralf Poscher, ‘Hermeneutics and Law’ in Michael N Forster and Kristin Gjesdal (eds), *The Cambridge Companion to Hermeneutics* (CUP 2018) 326; see also Ran Hirschl, ‘Comparative Methodologies’ in Masterman and Schütze (n 5) 11, 16.

11 As will soon be explained, the typology submitted here is not only to map the field of comparative constitutional studies, but also to bring the interpretive character of constitutional comparison to the fore. Obviously, my present typology is not the only way comparative constitutional studies are categorised. For exemplary typologies and the criteria in the field, see Hirschl, *Comparative Matters* (n 4) 232–44 (focusing on mode and method of inquiry); Hirschl, ‘Comparative Methodologies’ (n 10) 16–18 (in terms of ‘who is conducting the comparative constitutional inquiry, in what capacity and for what purpose’); Mark Tushnet, ‘The Possibilities of Comparative Constitutional Law’ (1999) 108 Yale LJ 1225 (identifying ‘functionalism’, ‘expressivism’ and ‘bricolage’ as the three ways of comparing constitutional experiences in relation to the interpretation of the United States Constitution) 1225, 1238–1306; Nicholas Aroney, ‘Comparative Law in Australian Constitutional Jurisprudence’ (2007) 26 UQLJ 317, 319–23 (focusing on the particularist–universalist spectrum of the scope of comparison); Vicki C Jackson, ‘Comparative Constitutional Law: Methodologies’ in Rosenfeld and Sajó (n 5) 54, 55–67 (categorising comparative constitutional scholarship according to classificatory, historical, normative, functional, and contextual methodological approaches); David S Law, ‘Introduction: Pedagogy and Conceptualization of the Field’ in David S Law (ed), *Constitutionalism in Context* (CUP 2022) 3, 5–18 (based on ‘models of pedagogy’); Rosalind Dixon, ‘Comparative Constitutional Modalities: Towards a Rigorous But Realistic Comparative Constitutional Studies’ (2024) 2 Comparative Constitutional Studies 60 (noting different comparative modes in terms of objects of comparison, such as constitutional history, argument, doctrine, practice and experience or value). Compared to the typologies above, my classification of comparative constitutional studies into four varieties is based on both objects of comparison and interpretive approaches.

12 For suggestions of ‘methodological pluralism’ and ‘analytical eclecticism’ in comparative constitutional studies, see Hirschl, *Comparative Matters* (n 4) 280 (quoting David S Law and Mila Versteeg, ‘The Evolution and Ideology of Global Constitutionalism’ (2011) 99 Ca L Rev 1163, 1248); cf Lorenzo Zucca, ‘Montesquieu, Methodological Pluralism and Comparative Constitutional Law’ (2009) 5 EuConst 481, 482–3, 497–500.

13 Here I borrow and adapt René David’s idea of legal families. See René David and John C Brierley, *Major Legal Systems in the World Today: An Introduction to the Comparative Study of Law* (2nd edn, Free Press 1978).

14 See Ming-Sung Kuo, ‘A Dubious Montesquieuian Moment in Constitutional Scholarship: Reading the Empirical Turn in Comparative Constitutional Law in the Light of William Twining and His Hero’ (2013) 4 TLT 487, 498–501; see also Donald R Kelley, *The Human Measure: Social Thought in the Western Legal Tradition* (Harvard UP 1990) 257–75, 279–80; Günther Frankenberg, *Comparative Law as Critique* (Edward Elgar 2016) 42–7, 54, 96–9.

15 Understood this way, methodology includes what Paul Kahn calls ‘the method of the methodless’ along with the conventional ones as discussed in the philosophy of science. For the method of the methodless and the importance of agenda framing in research, see Paul W Kahn, ‘Freedom and Method’ in Rob van Gestel, Hans-Wolfgang Micklitz and Edward L Rubin (eds), *Rethinking Legal Scholarship: A Transatlantic Dialogue* (CUP 2017) 499, 502–23. For a realist critique of methodology in the social sciences from the perspective of the philosophy of science, see Ian Shapiro, *The Flight from Reality in the Human Sciences* (Princeton UP 2005). Notably, the ‘social scientification’ of law and legal studies has met with criticisms. See Marianne Constable, ‘Genealogy and Jurisprudence: Nietzsche, Nihilism, and the Social Scientification of Law’ (1994) 19 L & Soc Inquiry 551; Marianne Constable, *Just Silences: The Limits and Possibilities of Modern Law* (Princeton UP 2005) 9–34. For a terminological clarification of ‘scientification’, see n 22.

16 Without evoking legal positivism in jurisprudence, ‘positivist’ here refers to what Marianne Constable has observed of ‘sociolegal positivism’: Constable, *Just Silences* (n 15) 28–34; see also Kelley (n 14) 268–77. For the classical meaning of positivism and its relationship with the social sciences, see Martin Hollis, *The Philosophy of Social Science: An Introduction* (rev edn, CUP 2002) 40–65.

17 Madhav Khosla, ‘Is a Science of Comparative Constitutionalism Possible?’ (2022) 135 Harv L Rev 2110; Ming-Sung Kuo, ‘The Reign of Constitutional Positivism: Revolution Reconceived in the New Constitutional Age’ (2022) 37 Constitutional Commentary 197; see also Kuo, ‘A Dubious Montesquieuian Moment’ (n 14); Or Bassok, ‘The World of Constitutionalism Is Not Flat’ (2023) 24 German Law Journal 686; cf Francesca Bignami, ‘Formal versus Functional Method in Comparative Constitutional Law’ (2016) 53 Osgoode Hall LJ 442, 443–5.

18 For how a constitutional phenomenon may fall from view, see Paul W Kahn, ‘Decision and Legal Interpretation’ in Miguel Nogueira de Brito and Luís Pereira Coutinho (eds), *The Political Dimension of Constitutional Law* (Springer 2020) 53, 60–1.

19 Compare Shapiro (n 15) with Constable, *Just Silences* (n 15). Situating jurisprudence and the social sciences in the Western intellectual tradition, Donald Kelley suggests that both have long been oriented towards the social order. Notably, ‘the social’ did not materialise as a field of knowledge until the social sciences took the place of legal scholarship and became the academic discipline specialising in understanding the social order. See Kelley (n 14); cf Tim Murphy, *The Oldest Social Science? Configurations of Law and Modernity* (OUP 1997) 77–153. I shall further discuss the implications of the social sciences to comparative constitutional studies as the argument unfolds.

20 Shapiro (n 15) 32–7.

21 Notably, some approaches discussed below reflect the interpretive turn in the human sciences. Seen in this light, ‘interpretation’ is hermeneutic without being absorbed into the hermeneutics. See Kelley (n 14) 279–83.

22 The scientific concept of knowledge is not confined to the research results of natural science; the social sciences generate knowledge by employing scientific methods, too. Nevertheless, given that the social sciences continue to be in the status of ‘proto-science’, the scientific character of the social sciences and their claim to be ‘scientific’ should be conditioned and considered accordingly. See Shapiro (n 15) 10–11, 17. Under the foregoing understanding, I apply the characterisation ‘scientific(ally)’ to the LS and documentary varieties of comparative constitutional studies throughout the article. When the social sciences pursue the scientific character without considering the fundamental difference between natural and social sciences, such a pursuit amounts to ‘scientification’ and is evocative of ‘scientism’.

23 The ‘Aims and Scope’ statement of the new journal *Comparative Constitutional Studies*—the inaugural issue of which was published in June 2023—illustrates this move: ‘*Comparative Constitutional Studies* (*CCS*) is an international journal for comparative public law scholarship’. Further information and statements about the journal are available at <www.elgaronline.com/view/journals/ccs/ccs-overview.xml?tab_body=about-copy1> accessed 17 January 2025. See also Hirschl, *Comparative Matters* (n 4) 151–90; but see Katherine G Young, ‘On What Matters in Comparative Constitutional Law: A Comment on Hirschl’ (2016) 96 BUL Rev 1375.

24 Hirschl, *Comparative Matters* (n 4) 20–76.

25 Casebooks on comparative constitutional law exemplify this variety of comparison. See Wen-Chen Chang and others, *Constitutionalism in Asia: Cases and Materials* (Hart Publishing 2014); Norman Dorsen and others, *Comparative Constitutionalism: Cases and Materials* (4th edn, West Academic Publishing 2022); Vicki Jackson and Mark Tushnet, *Comparative Constitutional Law* (3rd edn, Foundation Press 2014); see also Dieter Grimm, ‘Proportionality in Canadian and German Constitutional Jurisprudence’ (2007) 57 UTLJ 383; Niels Petersen, *Proportionality and Judicial Activism: Fundamental Rights Adjudication in Canada, Germany and South Africa* (CUP 2017).

26 In this article, I use the ‘constitution’ and define it broadly to include phenomena that are of fundamental significance to the functioning of the polity under examination besides the master-text constitution and constitutional law. In this sense, the constitution and the constitutional phenomenon as defined in section 1 are interchangeable. Thus, the concept of constitution includes both the ‘small-c constitution’ and the master-text constitution, too. Notably, while a country’s large-C Constitution is also its master-text constitution, there is no large-C Constitution in a country where the master-text constitution is entrenched but not codified. In the latter, the master-text constitution comprises all entrenched constitutional laws. Richard Albert, ‘The Difficulty of Constitutional Amendment in Canada’ (2015) 53 Alta L Rev 85, 112. That said, I use the two terms, master-text constitution and large-C Constitution (or simply Constitution), interchangeably. The legal constitution is part of the constitution, too. For the idea of the legal constitution, see András Sajó and Renáta Uitz, *The Constitution of Freedom: An Introduction to Legal Constitutionalism* (OUP 2017) 23–7. For a perceptive analysis of theories of small-c constitution, see Richard A Primus, ‘Unbundling Constitutionality’ (2013) 80 U Chi L Rev 1079, 1082–4, 1127–51.

27 Lawrence Lassig, *Fidelity & Constraint: How the Supreme Court Has Read the American Constitution Interpretation* (OUP 2019) 15–18.

28 Antonin Scalia, *A Matter of Interpretation: Federal Courts and the Law* (Princeton UP 1997).

29 ibid; Philip Bobbitt, *Constitutional Interpretation* (Blackwell 1991); Jed Rubenfeld, *Revolution by Judiciary: The Structure of American Constitutional Law* (Harvard UP 2005) 12–18.

30 Ronald Dworkin’s ‘Right Answer Thesis’ is the epitome of the association of the right answer with correct interpretation in constitutional scholarship. Compare Ronald Dworkin, *Taking Rights Seriously* (Harvard UP 1977) 279–90 with Ronald Dworkin, *Law’s Empire* (Belknap Press 1988) viii–ix, 229–32. See also James Q Whitman, ‘No Right Answer?’ in John Jackson, Maximo Langer and Peter Tilkers (eds), *Crime, Procedure and Evidence in a Comparative and International Context: Essays in Honour of Professor Mirjan Damaška* (Hart Publishing 2008) 371.

31 Jeremy Waldron, *‘Partly Laws Common to All Mankind’: Foreign Law in American Courts* (Yale UP 2012).

32 Examples include ibid; Tushnet, ‘The Possibilities of Comparative Constitutional Law’ (n 11); Frankenberg (n 14) 37–70; Cass R Sunstein, *A Constitution of Many Minds: Why the Founding Document Doesn’t Mean What It Meant Before* (Princeton UP 2009); Vicki C Jackson, *Constitutional Engagement in a Transnational Era* (OUP 2010) 103–16; Donald P Kommers, ‘The Value of Comparative Constitutional Law’ (2019) 20 German Law Journal 524.

33 David and Brierley (n 13).

34 Waldron calls the shared common reasoning among lawyers ‘legal episteme’. See Waldron (n 31) 93.

35 Sujit Choudhry (ed), *The Migration of Constitutional Ideas* (CUP 2006).

36 Waldron (n 31) 158–60.

37 ibid 160–1; see also Jakab, Dyevre and Itzcovich (n 5); David Robertson, *The Judge as Political Theorist: Contemporary Constitutional Review* (Princeton UP 2010).

38 See eg Christopher McCrudden, ‘Common Law of Human Rights? Transnational Judicial Conversations on Constitutional Rights’ (2000) 20 ojls 499; Doreen Lustig and JHH Weiler, ‘Judicial Review in the Contemporary World: Retrospective and Prospective’ (2018) 18 ICON 315, 336–7; Ming-Sung Kuo, ‘Whither Judicial Dialogue after Convergence? Finding Transnational Public Law in *Nomos*-Building’ (2021) 19 ICON 1536.

39 See eg Tushnet, ‘The Possibilities of Comparative Constitutional Law’ (n 11); Jackson (n 32).

40 See eg Cheryl Saunders, ‘The Use and Misuse of Comparative Constitutional Law’ (2006) 13 Indiana Journal of Global Legal Studies 37, 67; Michel Rosenfeld, ‘The Politics of Comparative Constitutional Inquiry: At the Crossroads of Law, Political Science, and Ideology’ (2016) 66 UTLJ 273, 276–7; see also Hirschl, *Comparative Matters* (n 4) 9.

41 See Hirschl, *Comparative Matters* (n 4); see also Rosenfeld (n 40) 277; Yash Ghai, ‘Sentinels of Liberty or Sheep in Woolf’s Clothing? Judicial Politics and the Hong Kong Bill of Rights’ (1997) 60 mlr 459, 479.

42 David S Law and Wen-Chen Chang, ‘The Limits of Global Judicial Dialogue’ (2011) 86 Wash L Rev 523, 535–68.

43 Hirschl, *Comparative Matters* (n 4); Jackson (n 32); Rosenfeld (n 40).

44 Michel Foucault’s noted concept of ‘power-knowledge’ is the prime example. See Michel Foucault*, Discipline and Punishment: The Birth of the Prison* (Alan Sheridan tr, Penguin Books 1991); Michel Foucault*, Security, Territory, Population: Lectures at the Collége de France, 1977–78* (Arnold I Davidson ed, Graham Burchell tr, Palgrave Macmillan 2007).

45 Jack M Balkin and Sanford Levinson, ‘Law and the Humanities: An Uneasy Relationship’ (2006) 18 Yale JL & Human 155, 179–80; see also Bruno Latour, *The Making of Law: An Ethnography of the Conseil d’Etat* (Marina Brilman and Alain Brilman Pottage trs, Polity 2010).

46 See Oran Doyle, ‘Constitutional Cases, Foreign Law and Theoretical Authority’ (2016) 5 GlobCon 85, 88–102. For present purposes, I leave aside the question of whether doctrinal comparison is the best tool for the acquisition of knowledge underpinning constitutional interpretation. See Tushnet, ‘The Possibilities of Comparative Constitutional Law’ (n 11).

47 Latour (n 45); Geoffrey Samuel, ‘Is Law Really a Social Science? A View from Comparative Law’ (2008) 67 CLJ 288, 308–13; cf Robert M Cover, ‘Violence and the Word’ (1986) 95 Yale LJ 1601. This observation is made in normal times. In exceptional times, such as revolutions, during which the court’s mandated final say is pushed aside, judicial pronouncements on constitutional principles no longer have claims to authority. See Kuo, ‘The Reign of Constitutional Positivism’ (n 17) 216–9.

48 Rosenfeld (n 40) 277–8; Doyle (n 46) 103–6.

49 See Robert M Cover, ‘The Supreme Court, 1982 Term—Foreword: *Nomos* and Narrative’ (1983) 97 Harv L Rev 4; see also Kuo, ‘Whither Judicial Dialogue’ (n 38).

50 See Robert Leckey, ‘Thick Instrumentalism and Comparative Constitutionalism: The Case of Gay Rights’ (2009) 40 Colum Hum Rts L Rev 425.

51 ibid 434–6.

52 See Kuo, ‘Whither Judicial Dialogue’ (n 38) 1541–57.

53 John P Dawson, *The Oracles of the Law* (University of Michigan Law School 1968).

54 See Waldron (n 31); see also Jakab, Dyevre and Itzcovich (n 5).

55 Waldron (n 31); see also Kuo, ‘Whither Judicial Dialogue’ (n 38) 1539–41.

56 Compare Kuo, ‘Whither Judicial Dialogue’ (n 38) 1549–57 with Rosalind Dixon, ‘Proportionality & Comparative Constitutional Law versus Studies’ (2018) 12 Law & Ethics of Human Rights 203.

57 See Paul W Kahn, *The Cultural Study of Law: Reconstructing Legal Scholarship* (University of Chicago Press 1999) 18–30; see also Latour (n 45); RC van Caenegem, *Judges, Legislators and Professors: Chapters in European Legal History* (CUP 1987); Mitchel de S-O-l’E Lasser, *Judicial Deliberations: A Comparative Analysis of Transparency and Legitimacy* (OUP 2004) 38–60; but cf Richard A Posner, *Divergent Paths: The Academy and the Judiciary* (Harvard UP 2016) 1–51; Robin L West, *Teaching Law: Justice, Politics, and the Demands of Professionalism* (CUP 2014) 131–73.

58 The Law and Society movement is formally associated with the establishment of the Law and Society Association in 1964. Susan S Silbey, ‘Law and Society Movement’ in Herbert M Kritzer (ed), *Legal Systems of the World: A Political, Social, and Cultural Encyclopedia, Volume II* (CUP 2002) 860, 861. The roots of Law and Society can be traced all the way back to Plato and Aristotle. See Brian Z Tamanaha, *A General Jurisprudence of Law and Society* (OUP 2001) 11–16; see also Howard (n 4) 5–6.

59 See Silbey (n 58) 860; see also Hirschl, *Comparative Matters* (n 4) 127; Kelley (n 14) 221.

60 See Zucca (n 12) 483, 498–9; George Klosko, *History of Political Theory: An Introduction: Volume II: Modern* (2nd edn, OUP 2013) 226–7; see also Hirschl, *Comparative Matters* (n 4) 130–1.

61 Hirschl, *Comparative Matters* (n 4) 127–30; Howard (n 4) 6; Zucca (n 12); Kelley (n 14) 221.

62 Felice J Levine, ‘“His” and “Her” Story: The Life and Future of the Law and Society Movement’ (1990) 18 Florida State University Law Review 69, 76; see also William Simon’s comment reproduced in Lawrence M Friedman, ‘The Law and Society Movement’ (1986) 36 Stan L Rev 763, 778.

63 Roscoe Pound, ‘The Need of a Sociological Jurisprudence’ (1907) 19 Green Bag 607.

64 Eugen Ehrlich, *Fundamental Principles of the Sociology of Law* (Walter L Moll tr, Harvard UP 1936).

65 Grant Gilmore, ‘Legal Realism: Its Cause and Cure’ (1961) 70 Yale LJ 1037.

66 Jennifer Hendry, ‘One Umbrella or Two? Comparative (Socio-)Legal Studies in Light of Globalisation’ (2021) Journal of Comparative Law 552, 555–7.

67 For the ideological split of legal realism into critical legal studies and law and economics, see Anthony T Kronman, *The Lost Lawyer: Failing Ideas of the Legal Profession* (Belknap Press 1993) 225–64.

68 The latest intervention is the Law and Political Economy (LPE) Project. See Jedediah S Purdy and others, ‘Building a Law-and-Political-Economy Framework: Beyond the Twentieth-Century Synthesis’ (2020) 129 Yale LJ 1784.

69 Hirschl, *Comparative Matters* (n 4) 166, 186–9.

70 See ibid 166–86.

71 ibid 151; see also Kelley (n 14) 239–51; cf Kronman (n 67) 185–209.

72 See Hirschl, *Comparative Matters* (n 4) 224–6.

73 Ran Hirschl, *City, State: Constitutionalism and the Megacity* (OUP 2020). The emergence of comparative constitutional studies that are characterised by their global reach will be further addressed in relation to documentary comparison.

74 See Niels Petersen and Konstantin Chatziathanasiou, ‘Empirical Research in Comparative Constitutional Law: The Cool Kid on the Block or All Smoke and Mirrors?’ (2021) 19 ICON 1810, 1811–13; see also Joanne McEvoy and Jennifer Todd, ‘Constitutional Inclusion in Divided Societies: Conceptual Choices, Practical Dilemmas and the Contribution of the Grassroots in Northern Ireland and the Republic of Ireland’ (2023) 58 Cooperation & Conflict 393 (employing methods of focus group study and interview).

75 Alexander Hudson, ‘Political Parties and Public Participation in Constitution Making: Legitimation, Distraction or Real Influence?’ (2021) 53 Comparative Politics 501.

76 See Kahn, ‘Freedom and Method’ (n 15) 508–9, 522–3; see also Kelley (n 14) 279–83.

77 See also Hendry (n 66) 561; Paul W Kahn, *Origins of Order: Project and System in the American Legal Imagination* (Yale UP 2019) 188–9.

78 Compare Zachary Elkins, Tom Ginsburg and James Melton, *The Endurance of National Constitutions* (CUP 2009) 2–3 with Hirschl, *Comparative Matters* (n 4) 227.

79 cf Barber (n 9) 83.

80 See eg Yvonne Tew, *Constitutional Statecraft in Asian Courts* (OUP 2020).

81 Hirschl, *Comparative Matters* (n 4) 166–91.

82 See eg Feng LIN, ‘Is It Constitutional to Demand Hong Kong Independence? A Comparative Study’ (2019) 49 HKLJ 507, 519–36.

83 See eg Nicholas Sambanis, ‘Partition as a Solution to Ethnic War: An Empirical Critique of the Theoretical Literature’ (2000) 52 World Politics 437; Donald L Horowitz, ‘The Cracked Foundations of the Right to Secede’ (2003) 14 (2) Journal of Democracy 5.

84 Hirschl, *Comparative Matters* (n 4) 186–91, 224–32.

85 See Kahn, *Origins of Order* (n 77) 4–5, 160–2.

86 A prime example is ‘The Comparative Constitutions Project’ <https://comparativeconstitutionsproject.org/> accessed 17 January 2025. Notably, there exists a subtype of documentary comparison that is focused on the classification of features as indicated in constitutional master texts rather than on the identification of general patterns or trends in constitutional design. Richard Albert’s rich study of formal constitutional change is a good example of classification-focused documentary comparison. Richard Albert, *Constitutional Amendments: Making, Breaking, and Changing Constitutions* (OUP 2019). My discussion of the documentary variety of comparative constitutional studies focuses on its emphasis on the identification of patterns and its implications.

87 Although the methods employed by documentary comparison may apply to other constitutional documents such as court judgments, my discussion of the documentary variety of comparative constitutional studies is focused on those centring on large-C Constitutions to bring forth its features.

88 David S Law and Mila Versteeg, ‘Sham Constitutions’ (2013) 101 Ca L Rev 863; see also Adam Chilton and Mila Versteeg, *How Constitutional Rights Matter* (OUP 2020).

89 Tom Ginsburg, Daniel Lansberg-Rodriguez and Mila Versteeg, ‘When to Overthrow Your Government: The Right to Resist in the World’s Constitutions’ (2013) 60 UCLA L Rev 1184.

90 Law and Versteeg (n 12).

91 Elkins, Ginsburg and Melton (n 78).

92 Adam Chilton and Mila Versteeg, ‘Rights Without Resources: The Impact of Constitutional Social Rights on Social Spending’ (2017) 60 Journal of Law and Economics 713.

93 David S Law and Mila Versteeg, ‘The Declining Influence of the United States Constitution’ (2012) 87 NYU L Rev 762.

94 Law and Versteeg (n 12).

95 Ginsburg, Lansberg-Rodriguez and Versteeg (n 89).

96 Law and Versteeg (n 88).

97 See Lawrence B Solum, ‘Constitutional Texting’ (2007) 44 San Diego L Rev 123, 126–9, 148–50.

98 See eg Law and Versteeg (n 12) 1202–26; see also Anne Meuwese and Mila Versteeg, ‘Quantitative Methods for Comparative Constitutional Law’ in Maurice Adams and Jacco Bomhoff (eds), *Practice and Theory in Comparative Law* (CUP 2012) 230, 245–55.

99 Law and Versteeg (n 88) 888–9.

100 See eg David S Law, ‘Globalization and the Future of Constitutional Rights’ (2008) 102 Northwestern University Law Review 1277, 1298.

101 Kuo, ‘A Dubious Montesquieuian Moment’ (n 14) 495–6.

102 See eg Meuwese and Versteeg (n 98) 234–5; see also Hirschl, *Comparative Matters* (n 4) 225–6, 267–9.

103 See Meuwese and Versteeg (n 98) 244.

104 See ibid 243–4; see also Bruce Ackerman, *Revolutionary Constitutions: Charismatic Leadership and the Rule of Law* (Belknap Press 2019) 39–40.

105 Meuwese and Versteeg (n 98) 237–9; see also Kuo, ‘A Dubious Montesquieuian Moment’ (n 14) 494–5.

106 See Adam Chilton and Mila Versteeg, ‘Identifying Constitutional Law’ (7 December 2021) <https://ssrn.com/abstract=3980207> accessed 17 January 2025.

107 cf Meuwese and Versteeg (n 98) 240.

108 To Hirschl, both reflect an interest in causal questions in social sciences-informed comparative constitutional studies. Hirschl, *Comparative Matters* (n 4) 224–81. I am indebted to an anonymous reviewer for this point.

109 ibid 245–52; Law and Versteeg (n 12) 1211.

110 Meuwese and Versteeg (n 98) 239.

111 ibid 237–9; Elkins, Ginsburg and Melton (n 78).

112 Elkins, Ginsburg and Melton (n 78) 2–3.

113 Hirschl, *Comparative Matters* (n 4) 227; Meuwese and Versteeg (n 98) 240–52. Such patterns are a function of systemic ordering. See Kahn, *Origins of Order* (n 77) 188.

114 Meuwese and Versteeg (n 98) 235, 253; see also Hirschl, *Comparative Matters* (n 4) 177–9.

115 See eg Law and Versteeg (n 88) 888–9.

116 See Meuwese and Versteeg (n 98) 240.

117 See Mila Versteeg and Tom Ginsburg, ‘Measuring the Rule of Law: A Comparison of Indicators’ (2017) 42 L & Soc Inquiry 100.

118 Compare Hirschl, *Comparative Matters* (n 4) 227 with Elkins, Ginsburg and Melton (n 78) 2–3.

119 See Kuo, ‘A Dubious Montesquieuian Moment’ (n 14); Bassok (n 17); Khosla (n 17); see also Sujit Choudhry, ‘Method in Comparative Constitutional Law: A Comment on Law and Versteeg’ (2012) 87 NYU L Rev 2078.

120 Law and Versteeg (n 88) 888–9.

121 For a critical discussion of a documentary comparison’s basing objectivity claim on the United Nations-compiled information, see Kuo, ‘A Dubious Montesquieuian Moment’ (n 14) 498–9.

122 Meuwese and Versteeg (n 98) 252–4.

123 See Kuo, ‘A Dubious Montesquieuian Moment’ (n 14) 495–6.

124 See Joxerramon Bengoetxea, ‘Legal Institutions and the Comparison of Legal Cultures’ (2022) 12 Oñati Socio-Legal Series 1647, 1655–9; cf Kahn, ‘Freedom and Method’ (n 15) 499.

125 Clifford Geertz, *The Interpretation of Cultures: Selected Essays* (Basic Books 1973).

126 See Daniel Bonilla Maldonado, *Legal Barbarians: Identity, Modern Comparative Law and the Global South* (CUP 2021) 35–7; see also Kahn, *The Cultural Study of Law* (n 57) 34–86; Kahn, *Origins of Order* (n 77) ix–xiv, 3–5.

127 See Paul W Kahn, ‘The Constitution and United States’ Culture’ in Mark Tushnet, Mark A Graber and Sanford Levinson (eds), *The Oxford Handbook of the US Constitution* (OUP 2015) 1013, 1019–25; see also Bonilla, *Legal Barbarians* (n 126).

128 See Kahn, *The Cultural Study of Law* (n 57); see also Daniel Bonilla Maldonado, ‘The Cultural Analysis of Law: Questions and Answers with Paul Kahn’ (2020) 21 German Law Journal 284.

129 Cover, ‘The Supreme Court’ (n 49); see also Kuo, ‘Whither Judicial Dialogue’ (n 38) 1549. For a conception of *nomos* centred on the boundary and space at the core of its Greek origin, see Carl Schmitt, *The* Nomos of *the Earth in the International Law of the* Jus Publicum Europaeum (GL Ulmen tr, Telos Press 2003) 67–79.

130 See Kahn, *The Cultural Study of Law* (n 57) 40–65, 77–86; see also Bonilla, ‘The Cultural Analysis of Law’ (n 128) 285–7.

131 Kuo, ‘Whither Judicial Dialogue’ (n 38) 1552–7.

132 See also Paul W Kahn, ‘Comparative Constitutionalism in a New Key’ (2003) 101 Mich L Rev 2677, 2678–80.

133 Charles Taylor, *Modern Social Imaginaries* (Duke UP 2004); see also Charles Taylor, *A Secular Age* (Belknap Press 2007) 171–6. For discussions of constitutional orders with reference to social imaginary, see Kahn, ‘The Constitution and United States’ Culture’ (n 127) 1019–25; Zoran Oklopcic, *Beyond the People: Social Imaginary and Constituent Imagination* (OUP 2018); Alexander Latham-Gambi, ‘Political Constitutionalism and Legal Constitutionalism: An Imaginary Opposition?’ (2020) 40 ojls 737; Jiří Přibáň, *Constitutional Imaginaries: A Theory of European Societal Constitutionalism* (Routledge 2022); Ming-Sung Kuo, ‘The Path Towards Sovereign Territory: Reading China’s (Anti)Federal Idea Against Its Modern Territorial Constitutional Imaginary’ (2023) 21 ICON 510.

134 Compare Hirschl, *Comparative Matters* (n 4) 189, 199, 227 with Kelley (n 14) 265–9.

135 See Bonilla, *Legal Barbarians* (n 126) 166–72.

136 See ibid; Kahn, ‘Freedom and Method’ (n 15); Kahn, *Origins of Order* (n 77); Kahn, ‘The Constitution and United States’ Culture’ (n 127).

137 See Kuo, ‘Whither Judicial Dialogue’ (n 38) 1541–9.

138 See Kahn, *Origins of Order* (n 77) 188–9.

139 I owe this point to an anonymous reviewer.

140 See Kahn, ‘Freedom and Method’ (n 15) 499–503; Kahn, *The Cultural Study of Law* (n 57).

141 Isaiah Berlin, *The Hedgehog and the Fox: An Essay on Tolstoy’s View of History* (Henry Hardy ed, 2nd edn, Princeton UP 2014).

142 cf ibid 1.

143 See also Aroney (n 11) 320.

144 See eg Kahn, ‘The Constitution and United States’ Culture’ (n 127); see also Kahn (n 132) 2678.

145 See Kahn, *Origins of Order* (n 77) 2–4; Kahn, ‘The Constitution and United States’ Culture’ (n 127) 1019–25.

146 Rogers M Smith, *Stories of Peoplehood: The Politics and Morals of Political Membership* (CUP 2003); see also Cover, ‘The Supreme Court’ (n 49); Ming-Sung Kuo, ‘Politics and Constitutional Jurisgenesis: A Cautionary Note on Political Constitutionalism’ (2018) 7 GlobCon 75, 105–6.

147 cf Ariel Dorfman, *Death and the Maiden* (Nick Hern Books 1991). I learnt this from a seminar on ‘radical evil’ led by Paul Kahn at Yale Law School.

148 See generally ‘Special Issue, Law and Political Imagination: The Perspective of Paul Kahn’ (2023) 24 German Law Journal 619.

149 See Hirschl, *Comparative Matters* (n 4) 195.

150 For the relationship between area studies and international relations, see ‘Guest Section, Does International Relations Need Area Studies?’ (2020) 16(1) St Antony’s International Review 173; cf Widner (n 6) 740–1.

151 Paul Kahn’s comparison of how Europe and the United States see the relationship between legitimacy and justice is a good example. See Kahn (n 132).

152 See Hirschl, *Comparative Matters* (n 4) 280; cf Zucca (n 12) 482–3, 497–500.

153 cf Hirschl, *Comparative Matters* (n 4) 226–7.

154 See generally Steven Shapin, *The Scientific Revolution* (University of Chicago Press 1995).

155 See Balkin and Levison (n 45) 163–77.

156 For a discussion of how natural philosophy heralded modern sciences in the Scientific Revolution and was then displaced by modern sciences, see Nicholas Maxwell, ‘In Praise of Natural Philosophy: A Revolution for Thought and Life’ (2012) 40 Philosophia 705, 705–8.

157 ibid.

158 Constable, ‘Genealogy and Jurisprudence’ (n 15); cf Hirschl, *Comparative Matters* (n 4) 166–89, 279–84.

159 See Fernand Gobet and others, ‘Introduction: Scientific Discovery in the Social Sciences’ in Mark Addis and others (eds), *Scientific Discovery in the Social Sciences* (Springer 2019) 1, 1–2; see also Shapin (n 154) 15–64.

160 See Shapin (n 154) 69–106; cf Hanchen Wang and others, ‘Scientific Discovery in the Age of Artificial Intelligence’ (2023) 620 Nature 47.

161 For an interpretivist critique of social research, see Celine-Marie Pascale, *Cartographies of Knowledge: Exploring Qualitative Epistemologies* (Sage 2011) 21–4, 53–4.

162 See generally DC Phillips and Nicholas C Burbules, *Postpositivism and Educational Research* (Roman & Littlefield 2000).

163 Compare Kahn, ‘Freedom and Method’ (n 15) with Balkin and Levison (n 45) 177–82.

164 See Frankenberg (n 14) 227–33.

165 Compare Hirschl, *Comparative Matters* (n 4) 279 with Shapiro (n 15) 16–19.

166 See Kahn, ‘Freedom and Method’ (n 15); cf Frankenberg (n 14) 227–9.

167 See Kahn, *Origins of Order* (n 77) 5, 202.

168 For the question of causal chains, see Neil Gross, ‘The Structure of Causal Chains’ (2018) 36 Sociological Theory 343.

169 For the boundedness of social scientific explanations, see John Gerring, *Social Science Methodology: A Unified Framework* (2nd edn, CUP 2012) 64–6.

170 Compare Carol Harlow, ‘Public Law and Popular Justice’ (2002) 65 MLR 1, 3–6 with Aziz Z Huq, ‘Standing for the Structural Constitution’ (2013) 99 Va L Rev 1435, 1491–1508.

171 Compare Or Bassok, ‘How to Investigate the Social Imaginary’ (2012) 5 Jerusalem Rev Leg Stud 2 with Paul W Kahn, ‘Political Theology Defended’ (2012) 5 Jerusalem Rev Leg Stud 28.

172 See Armin von Bogdandy, ‘Comparative Constitutional Law as a Social Science? A Hegelian Reaction to Ran Hirschl’s Comparative Matters’ (2016) 49 Verfassung und Recht in Übersee 178.

173 For objectivity and the ideology of scientism, see Shapin (n 154) 162–3.

174 Compare Hirschl, *Comparative Matters* (n 4) 224–81 with Kahn, ‘Freedom and Method’ (n 15).

175 cf Pascale (n 161) 53–4; Jennifer C Greene, ‘Knowledge Accumulation: Three Views on the Nature and Role of Knowledge in Social Science’ in Wendy Luttrell (ed), *Qualitative Educational Research: Readings in Reflexive Methodology and Transformative Practice* (Routledge 2009) 63, 68.

176 See eg Kahn, ‘The Constitution and United States’ Culture’ (n 127); cf von Bogdandy (n 172).

177 See Bonilla, *Legal Barbarians* (n 126) 172; but cf Kahn, ‘Freedom and Method’ (n 15) 500, 511, 519.

178 See Kahn, ‘Freedom and Method’ (n 15) 506.

179 ibid 523.

180 See also Owen M Fiss, ‘Objectivity and Interpretation’ (1982) 34 Stan L Rev 739, 751.

181 Standing apart from the social sciences-informed constitutional comparison, the cultural variety evokes resistance to ‘scientific’ labelling. See Kahn, ‘The Constitution and United States’ Culture’ (n 127) 1027.

182 See Shapiro (n 15) 194–8.

183 See Frankenberg (n 14) 227–9. This is a result of the relational character of the constitutional phenomenon in political ordering. For the limitation of contemporary political science and its blindness to the relational character of politics, see Shapiro (n 15) 172–7.

184 The emergence of written constitutions in the late 18th century has been regarded as the ‘spur[]’ of the emergence of comparative constitutional law as a disciplinary field distinct from political theory *per se*. Rosalind Dixon and Tom Ginsburg, ‘Introduction’ in Ginsburg and Dixon (n 5) 1, 2.

185 For concerns over the positivist propensity in comparative constitutional studies, see Khosla (n 17) 2113–14; see also Kuo, ‘The Reign of Constitutional Positivism’ (n 17); cf Petersen and Chatziathanasiou (n 74).

186 See also Barber (n 9) 63; cf Constable, *Just Silences* (n 15) 28–44.

